# Short-Read Assembly of Full-Length 16S Amplicons Reveals Bacterial Diversity in Subsurface Sediments

**DOI:** 10.1371/journal.pone.0056018

**Published:** 2013-02-06

**Authors:** Christopher S. Miller, Kim M. Handley, Kelly C. Wrighton, Kyle R. Frischkorn, Brian C. Thomas, Jillian F. Banfield

**Affiliations:** Department of Earth and Planetary Science, University of California, Berkeley, California, United States of America; Argonne National Laboratory, United States of America

## Abstract

In microbial ecology, a fundamental question relates to how community diversity and composition change in response to perturbation. Most studies have had limited ability to deeply sample community structure (e.g. Sanger-sequenced 16S rRNA libraries), or have had limited taxonomic resolution (e.g. studies based on 16S rRNA hypervariable region sequencing). Here, we combine the higher taxonomic resolution of near-full-length 16S rRNA gene amplicons with the economics and sensitivity of short-read sequencing to assay the abundance and identity of organisms that represent as little as 0.01% of sediment bacterial communities. We used a new version of EMIRGE optimized for large data size to reconstruct near-full-length 16S rRNA genes from amplicons sheared and sequenced with Illumina technology. The approach allowed us to differentiate the community composition among samples acquired before perturbation, after acetate amendment shifted the predominant metabolism to iron reduction, and once sulfate reduction began. Results were highly reproducible across technical replicates, and identified specific taxa that responded to the perturbation. All samples contain very high alpha diversity and abundant organisms from phyla without cultivated representatives. Surprisingly, at the time points measured, there was no strong loss of evenness, despite the selective pressure of acetate amendment and change in the terminal electron accepting process. However, community membership was altered significantly. The method allows for sensitive, accurate profiling of the “long tail” of low abundance organisms that exist in many microbial communities, and can resolve population dynamics in response to environmental change.

## Introduction

Microbial communities respond to, and effect change on, surrounding geochemical conditions. Advances in community proteogenomics and transcriptomics have allowed for understanding the molecular basis of this interplay for some communities of interest [1]–[5]. However, most inferences of microbe-environment interactions are still made with molecular surveys of community-wide taxonomic affiliation. For many years, the phylogenetic marker gene of choice for such surveys has been the small subunit (SSU) ribosomal rRNA gene, due to its high conservation across the domains of life and the ability to PCR-amplify the sequences from complex communities with so-called “universal” conserved primers [6], [7]. Currently, both the SILVA and Greengenes SSU databases contain nearly half a million high-quality sequences that can be used to place genes from newly characterized communities in context [8], [9].

While tens to thousands of full-length rRNA gene sequences are collected via Sanger sequencing of cloned PCR products, hundreds of thousands to millions of short hypervariable fragments from this gene can be analyzed using 454 sequencing. Early studies inferred community composition with reads of approximately 100 bp [10]. Subsequent studies used longer reads, and sometimes targeted alternative hypervariable regions [11]–[13]. With 454 pyrosequencing of hypervariable regions for community characterization, care has to be taken to distinguish novel sequences from sequence variants introduced due to the high error rate [14]–[16].

In recent years, many groups have exploited the scale and economics afforded by hundreds of millions of Illumina reads to survey microbial community composition [17]–[24]. Typically, the strategy has been one borrowed directly from the initial 454-based surveys: PCR amplify one or more hypervariable regions of the SSU gene and use the short sequenced tags to infer phylogeny. Because of the short read lengths (typically 100–150 bp) and error rate, a read quality-filtering step is usually employed prior to identification of operational taxonomic units (OTUs). Caporaso et al. observed that, in a mock community, diversity was over-estimated unless confident sequences were observed at least 10,000 times in an experiment, a level that represented ≥0.01% of reads. [18]. Although many groups have been able to distinguish communities using single-end reads [18], [19], [22], others have attempted to correct errors by choosing sequencing primers so that paired end reads overlap, increasing overall length and quality in the overlapped region [17], [20], [21], [24]. However, as many as 40% to 50% of the reads cannot be unambiguously merged and are discarded [17], [20], though this depends on both the quality of the sequencing run and the stringency of filtering. Non-overlapping paired-end fragments can also provide a higher number of informative bases, at the expense of lower-quality read ends and a potentially more complicated downstream pipeline [23].

Although the use of hypervariable regions has been a necessary compromise for the use of “next-generation” sequencing in SSU-based surveys of microbial diversity, using shorter fragments introduces several analysis challenges. Placing these shorter fragments within the context of a phylogeny constructed from full-length sequences is non-trivial. Composition-based approaches such as the RDP classifier have been adapted for use with short fragments [25], [26], as have alignment-based approaches utilizing multiple sequence alignment to top BLAST hits [27] or hidden Markov models [28] in combination with a reference tree. The choice of a specific hypervariable region can affect both the accuracy and specificity of phylogenetic assignment [29] as well as estimates of overall diversity [30]. Comparing community composition across studies performed by sequencing different hypervariable regions warrants extra caution.

Here, we adapted a recently reported algorithm developed to reconstruct near full-length 16S rRNA genes from Illumina metagenomic sequences, EMIRGE [31], so that it now can be used to analyze large datasets generated when the entire sequencing allocation is applied to long amplicons. The approach provided depth and resolution of the community composition of three samples collected before and after an aquifer was biostimulated by acetate addition [32]. We find that the method is reproducible, produces accurate abundance estimates, and uncovers persistently high alpha diversity and phylogenetic novelty across all biological samples, despite acetate-induced perturbation of community membership.

## Materials and Methods

### PCR amplification and sequencing

DNA extracted from each of the three biological sediment samples was used as template for amplification of the 16S rRNA gene with the primers 27F (5′-AGAGTTTGATCCTGGCTCAG-3′) and 1492R (5′-GGTTACCTTGTTACGACTT-3′) [33]. For each sample, amplicons from a gradient PCR reaction were pooled and used as input to standard Illumina library preparation. After shearing amplicons to an expected average fragment size of 300 bp (actual range of mean insert size: 251 bp–299 bp), twelve libraries were prepared (4 for each biological sample). Each of 12 unique barcodes (referred to below as indices 01–12) consisting of 7 nucleotides were incorporated downstream of the read 1 and read 2 sequencing primers (Table S1). Sequencing on one lane of Illumina HiSeq 2000 followed standard protocols. Raw reads are available in the NCBI Sequence Read Archive (SRA054986). Further details are provided in Methods S1.

### Subsample dataset creation and EMIRGE assembly of full-length 16S amplicons

For each barcoded library, raw reads were sampled at random without replacement into four separate 1 million read subsamples (see Methods S1). For each subsample, reads that passed minimum length thresholds after quality trimming were input into an amplicon-optimized version of EMIRGE [31] for assembly into full-length genes. This code is freely available at https://github.com/csmiller/EMIRGE. Briefly, EMIRGE relies on a database of candidate 16S sequences for template-guided assembly. In each iteration of a modified expectation-maximization algorithm, reads are first aligned and probabilistically attributed to candidate 16S genes. Subsequently, candidate gene abundances and consensus sequences are adjusted based on this probabilistic read attribution. Reconstructed gene abundances are estimated at termination by utilizing the final probabilistic accounting of reads. EMIRGE was run for each subsample for 120 iterations with default parameters (–join_threshold = 0.97) designed to merge reconstructed 16S rRNA genes if candidate consensus sequences share ≥97% sequence identity in any given iteration. The starting candidate rRNA database was derived from version 102 of the SILVA SSU database [9], which was filtered to exclude sequences shorter than 1200 bp and longer than 1900 bp, and clustered with USEARCH [34] at 97% identity to remove similar sequences. Characters with ambiguous IUPAC codes were replaced with an allowed character in the set ACTG at random. Insert size and standard deviation for each library (given above) were estimated by an initial mapping of reads to this database. EMIRGE-reconstructed 16S rRNA sequences with an estimated abundance of 0.01% or greater were kept for further analysis.

### Community analysis of EMIRGE sequences

EMIRGE-reconstructed 16S rRNA consensus sequences were used as input into standard QIIME version 1.4.0 workflows [35] for community analyses. All sequences from all 48 subsample runs were collected in order of decreasing estimated abundance, and representative OTUs were picked by clustering these sequences at 97% identity with USEARCH. Because in each subsample EMIRGE created consensus sequences potentially grouping reads from related (≥97% identical) sequences, it is theoretically possible that some across-sample clusters represented lower-abundance sequences that were <97% identical. An adjusted OTU table, containing the expected number of reads per OTU per sample, was constructed based on the number of mapping reads per sample and the EMIRGE-estimated relative abundance of each OTU per sample. OTUs were aligned with PyNAST [36] using a Greengenes [8] reference alignment (gg_97_otus_4feb2011.fasta). The PyNAST alignment was filtered and a phylogenetic tree was built using FastTree v.2.1.3 [37] with default parameters. Taxonomy to the family level was assigned to each OTU with the RDP classifier trained with the same Greengenes database and using a confidence threshold of 0.8. For Figure S2, phylum-level assignments were made by using the phylum from the best BLAST hit to the SILVA SSU NR database, version 108. Complete linkage clustering of Euclidian distances of phylum abundance vectors was performed in R (www.r-project.org).

Diversity measures were calculated within QIIME. For rarefaction analyses, 1000 to 500 000 reads were sampled from the original OTU table (step size = 9980; 10 replicates per sample), and rarefied OTU tables were clipped to set all counts ≤20 to 0. Principal coordinates plots were made using pairwise Unifrac distance matrices after normalizing for sequencing effort by randomly sampling 500000 reads from the OTU tables. Analyses of the V3 region of EMIRGE sequences were performed in an analogous manner to that of full-length sequences, except that V3 regions of each EMIRGE sequence were first excised *in silico* using PrimerProspector [38] with the primers 341F (5′-CCTACGGGAGGCAGCAG-3′) and 518R (5′-ATTACCGCGGCTGCTGG-3′) [17]. Any number of mismatches in the primer sequences were allowed, even though regions with multiple primer mismatches might not amplify in an actual experiment, so that every EMIRGE sequence had a candidate V3 region extracted. Regions less than 100 bp or more than 225 bp were discarded as possible errors, leaving 56,755 extracted V3 regions for analysis (99.5% of all EMIRGE subsample sequences).

### Spike-in control experiment

For the spike-in experiment, DNA from the iron-reducing sample was re-extracted and re-amplified under the same PCR conditions with the same 27F and 1492R primers. For this sample, an Illumina sequencing library was prepared with the barcode internal to the sequencing adapter using standard Illumina protocols. Prior to shearing and library preparation, the amplification products were amended with 0.5% DNA by mass of amplified PCR product from a clone containing the 16S rRNA gene from *Leptospirillum ferrodiazotrophum* [39]. The amplicon sequence (GenBank accession: JX235335) was verified by Sanger sequencing from primers 27F and 1492R.

### Analysis of amplicon end bias

The bias of library fragment start sites for amplicon ends was analyzed for a representative subsample of EMIRGE-reconstructed 16S rRNA genes (index 2, subsample 3) by mapping reads using bowtie version 0.12.7 [40] with permissive parameters (−n 3 −l 15 −e 400). For each read pair, the starting position closest to an amplicon end was recorded, as was total per-base coverage. Calculations of expected coverage are given in Methods S1.

## Results

### Reconstruction of near-full-length 16S rRNA genes from aquifer communities

We collected sediment from a previously un-amended portion of the Rifle aquifer (Department of Energy Integrated Field Research Challenge Site, Colorado) and used this to seed columns incubated in drilled wells. The aquifer was amended with acetate, and columns were recovered at different time points. The first “background” sample was recovered prior to acetate amendment. A second sample was taken after amendment once the community had transitioned to iron-reduction as the dominant terminal electron accepting process (TEAP), and a third once sulfate reduction was the dominant TEAP. 16S rRNA gene amplicons from a total of 48 subsamples, representing the three biological samples and two levels of technical replication (see methods) were processed through the analysis pipeline (16 subsamples per biological sample; Table S1).

Near-full-length 16S rRNA gene sequences (median 1474 bp) were reconstructed with EMIRGE. Sequencing errors were handled by letting EMIRGE choose a most-probable consensus for each SSU sequence based on the coverage acquired from multiple reads per consensus base. Reads were trimmed and filtered for quality, resulting in a minimum of 686,114 and a maximum of 843,139 pairs input into EMIRGE per subsample (Table S1). Abundance estimates for each assembled 16S rRNA gene were derived by the probabilistic accounting in EMIRGE of how reads map to each assembled rRNA sequence [31].

**Figure 1:**
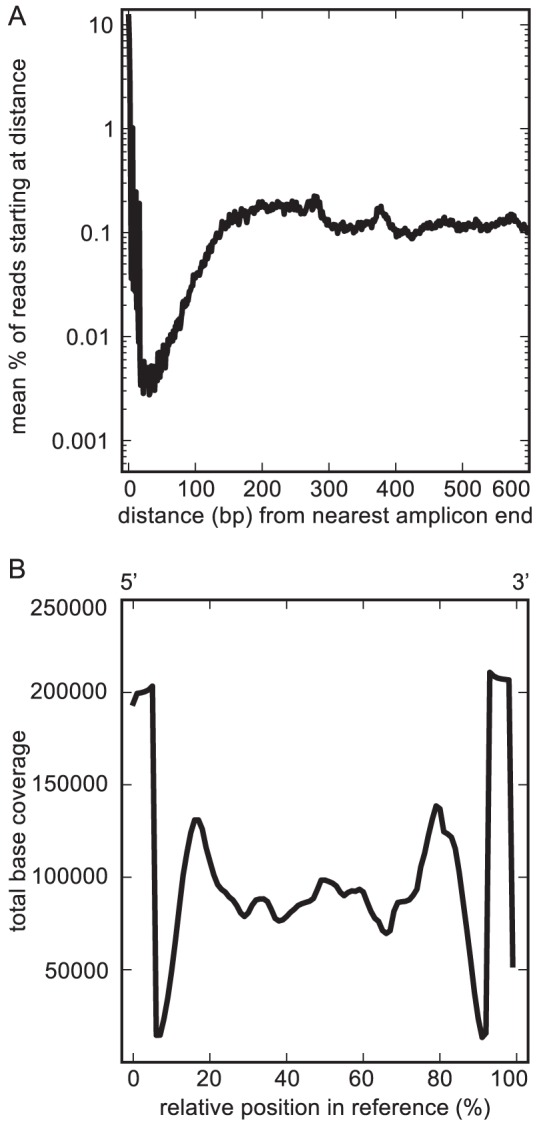
Sequencing bias for amplicon ends. Shown are data determined by mapping reads for a representative library (index 2 subsample 3) against EMIRGE-reconstructed16S rRNA sequences. **A** Proportion of mapped library fragments (y-axis) that begin a given number of bp away from the nearest reconstructed amplicon end (x-axis), averaged across all reconstructed 16S rRNA amplicons. There is a strong preference for fragments to begin at position 0 or 1. **B** Total library base coverage plotted in terms of relative position within an amplicon. Average reconstructed amplicon length was 1464 bp.

Reads were not distributed evenly across the length of reconstructed full-length gene sequences (Figure 1), an effect previously seen with Illumina sequencing of amplicons [41], [42]. Instead, on average, read pairs were approximately 100 times more likely to have one read begin at an amplicon end than at a position in the middle of an amplicon (Figure 1a). However, reads were unlikely to start near, but not at, the ends of amplicons, and thus the per-base coverage bias was not as pronounced (Figure 1b). With this positional bias, a sequence covered by 100 reads in a library of one million 93 base-pair reads (0.01% relative abundance) should have a base coverage of ∼11 X in non-end regions of the sequence, and >98.5% of reconstructed bases should have at least 5 X coverage.

### Community structure as revealed by EMIRGE

We focused our analyses on reconstructed sequences with a relative abundance of 0.01% or greater. Below a value in this range the expected sequence coverage drops to an unacceptably low level (see above). For the background samples, a mean of 1217 OTUs were reconstructed, while for the iron-reduction and sulfate-reduction samples, a mean of 1195 and 1154 were reconstructed, respectively (Table S1). Compared to other background samples, index 01 did not behave as anticipated (discussed below), and under-represented richness in four of the background subsamples. If these four subsamples are removed, the remaining 12 background samples had a mean of 1252 OTUs.

We used standard QIIME [35] workflows to further process the full-length sequences, assign taxonomy, and measure community diversity. EMIRGE consensus sequences from all subsamples with estimated abundance > =  0.01% were first clustered at 97% identity into OTUs, resulting in 46,223 OTUs that appeared in at least one subsample (where each OTU in each subsample was assembled from multiple reads). We classified as high-specificity those OTUs identified in ≥12 of the 16 subsamples for one or more biological sample. Using this definition, we identified 187 such “high-specificity” OTUs, which represented on average 40%, 42%, and 47% of the cumulative estimated relative abundance in the background, iron-reducing, and sulfate-reducing communities. More abundant OTUs tended to also be higher confidence, appearing in more replicate samples (Spearman rank correlation = 0.49; p-value 2.9e-56).

**Figure 2:**
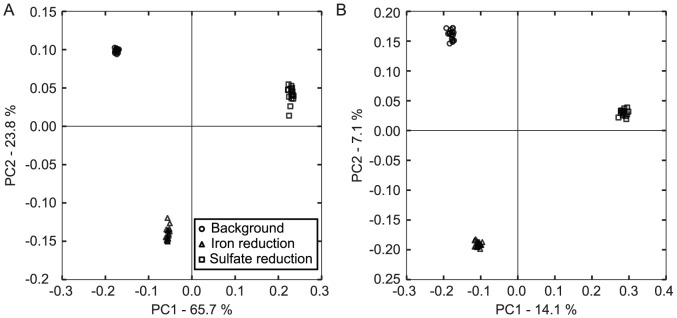
Principal coordinates analysis clusters the 48 subsample communities by biological sample. EMIRGE-reconstructed rRNA genes were used to construct a phylogenetic tree. From this tree, pairwise distances were calculated between each of the 48 subsample communities using either abundance-weighted (**A**) or unweighted (**B**) Unifrac, and principal coordinates analysis was used to reduce the dimensionality of the resulting distance matrices for visualization. Percentage variation explained by each principal coordinate is shown for each axis. Subsample communities clearly separate by biological sample. Weighted Unifrac accounts for a larger fraction of the variance in the first two principle coordinates than unweighted unifrac, indicating that changes in abundances are particularly informative.

Beta (between sample) diversity measurements calculated with pairwise weighted and unweighted Unifrac [43] distances indicated high similarity within the 16 samples from each biological replicate (Figure 2). With unweighted Unifrac, which is more sensitive to total richness, principal coordinates analysis revealed that principal coordinate 3 clearly separated subsamples with index 01 from other background samples (Figure S1). This bias was not apparent when considering weighted Unifrac distances, and was not observed for other indices. Thus, EMIRGE-reconstructed full-length 16S rRNA sequences are sufficient to distinguish among distinct biological communities, and this ability is largely independent of any variability introduced by library preparation or potential sampling artifacts introduced by the algorithm.

**Figure 3:**
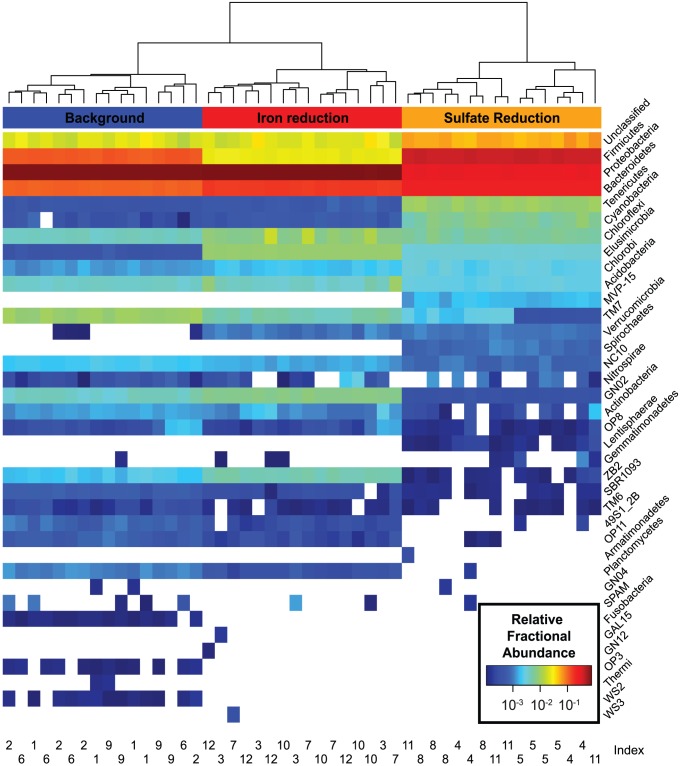
Phylum-level abundances of the 48 EMIRGE-reconstructed communities. Taxonomic assignments were made with the RDP classifier for each OTU with a confidence cutoff of 0.8, and abundances were summed to the phylum level and are shown as a log-scaled heatmap. The barcoding index for each sample is listed along the bottom. Hierarchical clustering of the abundance vectors separates each community by biological sample.

As an alternative to Unifrac, which uses an explicitly built phylogenetic tree, we also used the RDP classifier to taxonomically classify EMIRGE-generated 16S rRNA sequences based on shared short words with a reference training taxonomy [25]. When phylum-level abundance vectors are hierarchically clustered, subsamples group clearly by biological sample, and there is again little evidence to suggest that subsamples instead cluster by barcode index (Figure 3). Technical replicates are highly similar to each other. The Pearson correlation between phylum-level abundances from the same biological sample was 0.9998+/−0.0003 (mean +/− standard deviation). For comparison, between-biological-sample correlation at the phylum level was 0.7686+−0.1590. High correlation was also observed for within-biological-sample replicates when considering family-level abundances, the most specific taxonomic level assigned by the standard QIIME pipeline (Pearson r = 0.9920+−0.0071; r = 0.4936+−0.0976 for between-biological-sample replicates). We also performed the same analysis by assigning taxonomy via the best blast hit to the SILVA nonredundant rRNA database [9]. Although some phyla only were assigned with one taxonomic method, due to differences in the underlying reference databases, overall abundance patterns and reproducibility were similar (Figure S2).

**Figure 4:**
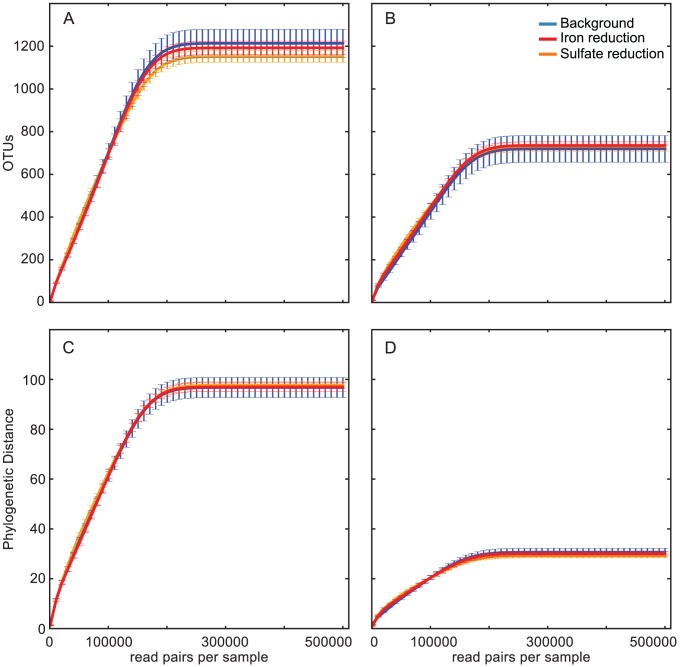
Alpha diversity of communities inferred by full-length rRNA and hypervariable-regions. Alpha diversity metrics are shown for EMIRGE-reconstructed full-length OTUs (**A, C**) and OTUs based on *in silico*-extracted V3 regions from the EMIRGE-reconstructed sequences (**B, D**). **A** and **B** show the total number of OTUs identified with increasing sequencing effort. **C** and **D** show the total tree phylogenetic distance (PD) observed with increasing sequencing effort. Plots show the mean and standard deviation of 10 samples per simulated sequencing effort. Blue: background; red: iron reduction; orange: sulfate reduction.

To assess alpha diversity, we recorded both the total number of OTUs (>0.01% abundance) and the total phylogenetic distance (PD), or branch length, in the phylogenetic tree per subsample. We performed a modified form of rarefaction to infer at what level of sequencing we could have observed the same number of OTUs or PD per sample. The number of observed species and PD plateaus quickly with increasing sampling of expected reads (Figure 4 a,c). This rarefaction analysis indicates that the diverse communities observed here could be recovered from roughly 200,000 paired-end reads. While additional sampling beyond this limit may be theoretically redundant, such rarefaction analyses assume that reads can properly be assigned to OTUs. In the case of EMIRGE, additional reads strengthen the confidence of the reconstructed sequences and abundance estimates.

Another way of inferring how close EMIRGE is to reconstructing all rare variants in a sample is to determine the fraction of reads successfully mapped to EMIRGE-generated sequences. If EMIRGE has faithfully reproduced the SSU sequences present, then all high-quality reads should map to one of the reconstructed SSU genes. For the 48 technical replicates, after the final algorithm iteration, 81.4% to 86.1% of reads mapped to at least one reconstructed SSU sequence (Table S1), indicating that most of the community diversity is likely captured with the depth of sequencing used here.

We queried how effective EMIRGE was at recovering a known sequence from a complex community. DNA from the iron-reduction sample was re-extracted and re-sequenced after spiking with a known amount of 16S amplicon from a species (*Leptospirillum ferrodiazotrophum*) not previously detected in the sample. We verified that the community profile from this EMIRGE run with spike-in control was similar to that of the original iron-reducing sample. Phylum level abundances correlated well (Pearson correlation 0.999), with one of the more notable discrepancies due to the spike-in control (Figure S3). A single sequence was reconstructed for the spike-in control species, as expected. This sequence was estimated to have a relative abundance of 0.21%, slightly less than the 0.50% by DNA mass spiked in to the library preparation. Except for two 1 bp indels (EMIRGE does not handle indels), the reconstructed sequence was identical to the expected amplicon sequence.

### Comparison of full-length SSU sequences to short hypervariable regions

Several groups have attempted to overcome the short read lengths and increased-3′-end error rates of Illumina hypervariable region sequencing by choosing primers so that paired-end reads overlap [17], [20], [21], [24]. We used the same primers as Bartram et al [17] to extract *in silico* the ∼150 bp V3 regions contained in the EMIRGE-generated full-length sequences, and asked how these shorter regions described community diversity. This analysis did not consider the substantial errors associated with raw paired sequencing reads [20], but instead assumed that perfect overlap and recovery of V3 regions was possible.

Utilizing the V3 region for community characterization increased the number of unclassified OTUs, and underestimated the alpha diversity of the three microbial communities. Across all samples, using just the V3 region as opposed to the full length sequences increased the percentage of unclassified OTUs at the phylum level from 8.6% to 34.6%. Even when allowing more error-prone assignments with a relaxed RDP classifier confidence threshold (0.5 instead of 0.8), the percentage of V3 OTUs with unclassified phyla is still high (16.6%). The replicate samples still clustered by sample type when weighted or unweighted Unifrac was used to measure between-sample differences (Figure S4). However, measured alpha diversity was decreased when using just the V3 region, with the number of observed OTUs roughly 60% of that observed with full-length sequences (Figure 4b), and PD approximately 1/3 the level measured with full-length sequences (Figure 4d).

### Community shifts accompanying changes in terminal electron accepting processes

**Figure 5:**
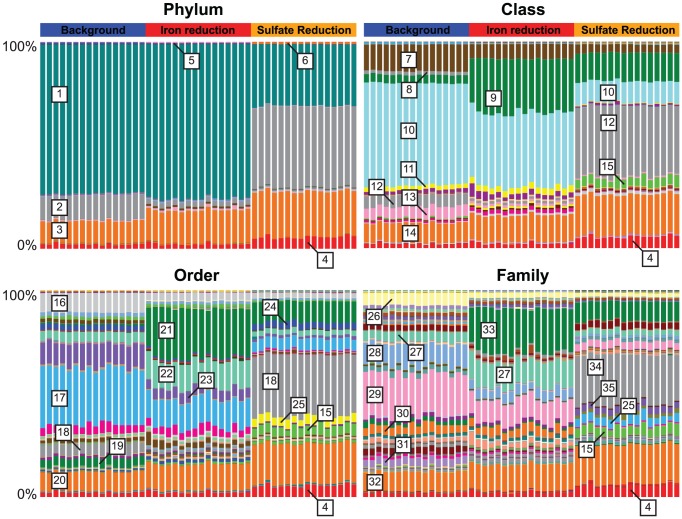
Community structure at varying levels of taxonomic resolution. Reconstructed full-length OTUs were assigned taxonomy by the RDP classifier, and relative abundances at 4 taxonomic levels are shown for each of the 48 subsample datasets. Indices from left to right in each panel are as in Figure 2. Select taxa are identified: 1. Proteobacteria, 2. Firmicutes, 3. Bacteroidetes, 4. Unassigned, 5. TM7, 6. Tenericutes, 7. Gammaproteobacteria, 8. Epsilonproteobacteria, 9. Deltaproteobacteria, 10. Betaproteobacteria, 11. Alphaproteobacteria, 12. Clostridia, 13. Bacilli, 14. Bacteroidia, 15. Unclassified Firmicute, 16. Pseudomonadales, 17. Burkholderiales, 18. Clostridiales, 19. Bacillales, 20. Bacteroidales, 21. Desulfuromonadales, 22. Rhodocyclales, 23. Methylophilales, 24. Desulfobacterales, 25. Unclassified Clostridia, 26. Pseudomonadaceae, 27. Rhodocyclaceae, 28. Methylophilaceae, 29. Comamonadaceae, 30. Unclassified Betaproteobacteria, 31. Bacillaceae, 32. Unclassified Bacteroidales, 33. Geobacteraceae, 34. Peptococcaceae, 35. Unclassified Clostridiales.

At all levels of taxonomic resolution, there were important differences in community composition among the background, iron-reducing and sulfate-reducing sediments (Figure 5). At the phylum level, the change from unstimulated to iron-reducing community was subtle. However, certain families become markedly more or less abundant upon stimulation, despite overall similar alpha diversity. For example, there is a clear increase in *Geobacteraceae*, which are barely present in the background (0.65%) but make up roughly 21% of the iron-reducing community. This is consistent with previous studies showing dominance of this family in the planktonic phase of the aquifer under acetate-stimulated iron reduction. [32], [44]–[47]. When we examined specific EMIRGE sequences within the *Geobacteraceae*, we found evidence for a strong response for specific species. For example, EMIRGE OTU 37084 increased from 0.2% of background sequences to 6.6% of iron-reduction sequences. This OTU shares 97% sequence identity with *Geobacter bemidjiensis* Bem, an organism emblematic for subsurface iron reduction [48].

In the sulfate reducing community, phylum-level differences in abundance were pronounced (Figures 3 and 5); most notable was a sharp increase in the number of *Firmicutes* detected, often closely related to known sulfate reducing taxa. The family *Peptococcaceae*, present as 1.6% and 0.3% of the background and iron-reducing communities, make up 23.8% of the sulfate-reducing community. Some high-specificity, high-abundance EMIRGE sequences represented known sulfate-reducing bacteria (e.g. OTU 9461, 98% identical to *Desulfosporosinus* species and 3.3% abundance). However, we also recovered sequences representing potentially novel and important sulfate reducing species. For example, OTU 2554 was not detectable in the background sample, but was reconstructed in the sulfate-reducing community at a relative abundance of 8.3%. This sequence shares only 95% sequence identity to its closest BLAST hit, *Desulfotomaculum acetoxidans*, a sulfate-reducer known to grow on acetate [49]. Thus, both in the iron-reducing and sulfate-reducing communities, the method captured known biological responses to environmental change at the species level.

In addition to detecting organisms consistent with known biological responses to a shift to iron or sulfate reduction, we also observed many sequences from phyla with few or no cultured representatives (Figure 3). For example, OTUs classified as candidate division TM7 make up ∼1% of the background community and drop in relative abundance by an order of magnitude by the time the community has transitioned to sulfate reduction. Candidate division BD1-5 organisms (classified as GN02 by the RDP classifier) also exist at low levels in the background and iron-reducing samples (0.4% and 0.7%), and relative abundances drop ten-fold in sulfate reduction (0.04%). One of these BD1-5 sequences (OTU 39774) makes up 0.2% of the background community and shares only 89% identity to the nearest environmental clone (there are no isolates from this phylum). Sequences related to candidate division OD1 (Figure S2) consistently make up approximately 0.6% of each community.

## Discussion

The ability to explore microbial community composition and detect rare members provides the opportunity to develop a better understanding of how microorganisms are distributed within and across different ecosystem types. This may be important, for example, when seeking to characterize the environmental repositories of pathogens [50]–[52] or to infer functional capacity, such as nutrient cycling [53], [54]. Profiling of microbial diversity in a way that extends detection far out on rank abundance curves (Figure S5) in spatial or temporal samples makes it possible to understand how different resources or physical/chemical conditions impact ecosystem structure. Such methods can constrain organism sources and may provide clues to the physiology of rare organisms. These insights may be important, for example, in studies of infant gut colonization [55], [56] or bioremediation [45].

Here, we applied an updated version of the EMIRGE algorithm to investigate microbial communities in sediment before and after perturbation. Because EMIRGE reconstructs essentially full-length sequences, we achieved sufficient taxonomic resolution to detect how specific organisms responded to altered conditions. We show proliferation of organisms that, through correlation of their relative abundances with geochemical measurements, likely contribute the biochemical functionality that accounts for observed conditions. For example, iron- and sulfate-reduction processes are likely linked to proliferation of *Geobacteraceae* and *Peptococcaceae*, respectively. Although the role of these families in iron- and sulfate-reduction is well documented, the ability to resolve which specific species are responsible may have broader implications. For example, such linkages can be incorporated into reactive transport models that attempt to describe the overall coupling of biological and geochemical processes [32], [57]. We also detect many rare members from uncultivated phyla. The roles these bacteria play in subsurface geochemistry is only beginning to be elucidated [58].

Our analyses document persistently very high biodiversity in acetate-amended sediment. In the single timepoints sampled during iron and sulfate reduction, we do not detect strong proliferation of a few organisms in response to acetate stimulation, contrary to results of prior clone-based studies of the Rifle aquifer [44], [46] but consistent with a deep community profiling by PhyloChip microarray [45]. Beyond methodological differences in sensitivity, aquifer geochemical heterogeneity has been documented and shown to affect acetate availability and community composition during secondary stimulation [59]. The aquifer has a wide grain size distribution, and a variety of carbon substrates are likely in the sediment due to varying Colorado River riparian zone inputs at the time of sediment deposition. Other factors, such as increased resource complexity due to microbial processes (breakdown of refractory organic carbon, production of sulfide, hydrogen, etc.) may contribute the wide niche variety required to maintain high microbial diversity. Alternatively, our time points may have simply missed organism blooms.

EMIRGE has potential advantages over sequencing of short hypervariable regions. The increased length provided by full-length sequences has the potential to provide a more detailed taxonomic description of microbial communities. Although some studies show short rRNA hypervariable regions track full-length gene taxonomies well, there are conflicting reports of which hypervariable region is most suitable [29], [60] and how reproducible the method is [61], [62]. Short regions are also useful for the simpler task of discriminating among biologically distinct communities [63]. However, we find that using just the V3 portion of the full-length sequences reconstructed here significantly decreases the number of sequences we can assign to specific taxa, and also decreases the apparent phylogenetic diversity within a community (Figure 4), a result consistent with previous simulation studies [30]. With an assembly-based strategy that utilizes multiple reads to assign each base in a consensus sequence, EMIRGE also aims to eliminate the “false” rare biosphere associated with increased error of newer sequencing technologies [15], [16]. Remarkably, even with the highest stringency quality controls that discarded 97% of the reads, one careful Illumina-based study that sequenced the V6 region of a single *Escherichia coli* culture with two 16S rRNA gene copies recovered 775 different tag sequence OTUs, many with abundances >0.01% [20]. In the current study, EMIRGE reported exactly one correct sequence from a spike-in control species, highlighting the utility of dealing with sequencing error via an assembly-based strategy.

There are also limitations to the approach described here. Like all 16S-rRNA gene based surveys, EMIRGE measures relative abundances of genes, not organisms. Organism-specific differences in gene copy number can alter the apparent abundance of community members and lead to false conclusions about community structure [64]. There is evidence that, through selection, average copy number in a community may fluctuate in response to environmental change or during succession, further obfuscating measures of relative abundance [65]. PCR bias associated with different primers or sequence composition can result in underrepresentation or overrepresentation of certain clades [66]. Relative abundances can also be misleading if total cell numbers change dramatically via growth or death of certain lineages. A modification to experimental protocols that quantified absolute cell or DNA abundance could assist with distinguishing relative vs. absolute changes. In contrast to techniques that incorporate barcodes directly in PCR primers, the current EMIRGE protocol requires that each sample is prepared and sheared as a separate library. Thus, library preparation cost, while continuing to decrease, can be a limiting factor, and EMIRGE may be most beneficial for studies utilizing Illumina's lower-throughput MiSeq instrument. Finally, because of the shearing step, overrepresentation of amplicon ends consumes sequencing unnecessarily (Figure 1), a problem that may be mitigated with changes to library preparation protocols [41].

The sediment biosphere is largely unknown, despite its massive volume, high importance as a reservoir of cells and nutrients [67] and, as shown here, high phylogenetic diversity. Organisms in the subsurface, such as in aquifer sediments, play important ecosystem roles. Impacts may range from local control of contaminant, carbon, and other compound cycling to health effects due to influence on water quality (e.g., as a reservoir of pathogens of humans, animals, agricultural pests) to global carbon cycle consequences through transformations of buried refractory organic carbon compounds and methane. Analyses presented here provide a first illustration of how a high throughput sequencing method with low systematic errors combined with full-length reconstruction of the widely sampled and phylogenetically informative 16S rRNA gene can aid in our understanding of these topics.

## Supporting Information

Figure S1 **Principal coordinates analysis highlighting community differences by sequencing library barcoding index.** EMIRGE-reconstructed rRNA genes were used to construct a phylogenetic tree using v.2.1.3 with default parameters. From this tree, pairwise distances were calculated between each of the 48 subsample communities using unweighted Unifrac as in Figure 2. Principal coordinates analysis was used to reduce the dimensionality of the resulting distance matrix for visualization. With unweighted Unifrac as the distance metric, Principal coordinate 3 clearly separates Index 1 away from the other background samples, although this only explains 2.2 percent of the variation.
(EPS)

Figure S2 **Phylum-level abundances of the 48 EMIRGE-reconstructed communities as assigned by SILVA BLAST.** Taxonomic assignments were made by adopting the phylum of the single best blast hit to the SILVA SSURef 108 rRNA database for each OTU, and abundances were summed to the phylum level and are shown as a log-scaled heatmap. Barcoding index for each sample is listed along the bottom. Hierarchical clustering of the abundance vectors separates each community by biological sample.
(EPS)

Figure S3 **Phylum-level abundance concordance between iron-reducing samples and re-extracted spike-in control.** Phylum-level relative abundances were calculated for the spike-in control iron-reducing sample and a representative subsample (index 3 subsample 3), and are plotted on a log scale. Each square is an individual phylum, and Nitrospira, the phylum of the spike in control (added at 0.005 relative abundance), is indicated with an open circle. Pearson correlation = 0.999.
(EPS)

Figure S4 **Principal coordinates analysis using V3 regions of the 48 subsample community reconstructions.** V3 regions were extracted from EMIRGE-reconstructed rRNA genes, and these regions were used to construct a phylogenetic tree. From this tree, pairwise distances were calculated between each of the 48 subsample communities using either abundance-weighted (**a**) or unweighted (**b**) Unifrac, and principal coordinates analysis was used to reduce the dimensionality of the resulting distance matrices for visualization. Percentage variation explained by each principal coordinate is shown for each axis. Subsample communities clearly separate by biological sample.
(EPS)

Figure S5 **Rank abundance curves for the 48 technical replicates.** All OTUs are plotted on a log scale, and the relative abundance cutoff of 0.01% is shown with a horizontal line. Inset: zoom of first 40 OTUs per sample, plotted on a linear scale to highlight similarity in community structure among the most abundant OTUs.
(TIF)

Table S1 **Description of the 48 data sets analyzed with EMIRGE.**
(DOC)

Methods S1 **Additional details of sample collection, DNA extraction, amplification, sequencing, and analysis.**
(DOC)
