# Supplementary figures and images for: Short-Read Assembly of Full-Length 16S Amplicons Reveals Bacterial Diversity in Subsurface Sediments
Source: PLoS One. 2013 Feb 6;8(2):e56018. doi: 10.1371/journal.pone.0056018 (PMC3566076; doi:10.1371/journal.pone.0056018)

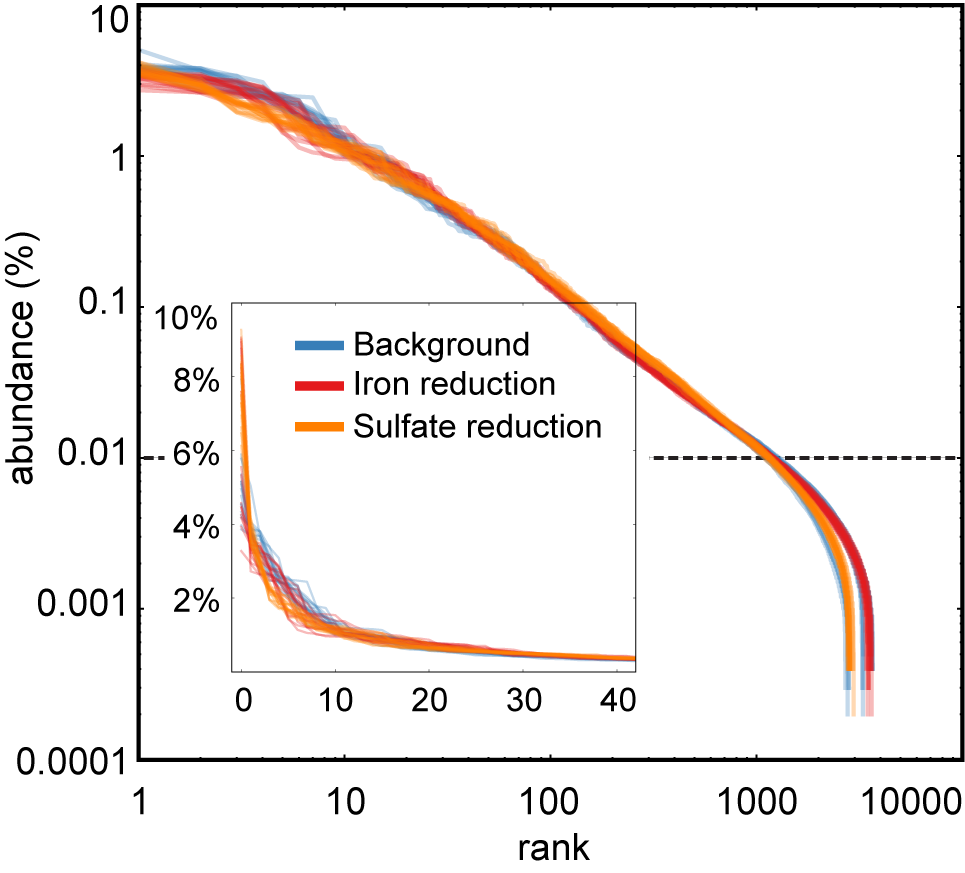

Supplement: Figure S5 — Rank abundance curves for the 48 technical replicates. All OTUs are plotted on a log scale, and the relative abundance cutoff of 0.01% is shown with a horizontal line. Inset: zoom of first 40 OTUs per sample, plotted on a linear scale to highlight similarity in community structure among the most abundant OTUs. (TIF) [file pone.0056018.s005.tif]
