# Supplementary material for: Short-Read Assembly of Full-Length 16S Amplicons Reveals Bacterial Diversity in Subsurface Sediments
Source: PLoS One. 2013 Feb 6;8(2):e56018. doi: 10.1371/journal.pone.0056018 (PMC3566076; doi:10.1371/journal.pone.0056018)
Supplement: Table S1 — Description of the 48 data sets analyzed with EMIRGE. (DOC) [file pone.0056018.s006.doc]

**Table S1. Description of the 48 data sets analyzed with EMIRGE**

| Biological Sample | Library Index | Index Sequence | Sub-sample | Trimmed Read Pairs | Pairs  Mapped (%) | OTUs | PD |
| --- | --- | --- | --- | --- | --- | --- | --- |
| Background | 1 | CACAGTT | 0 | 826,054 | 699,992 ( 85 ) | 1,103 | 90.9 |
| Background | 1 | CACAGTT | 1 | 825,727 | 701,219 ( 85 ) | 1,096 | 89.2 |
| Background | 1 | CACAGTT | 2 | 825,778 | 700,245 ( 85 ) | 1,115 | 89.4 |
| Background | 1 | CACAGTT | 3 | 825,813 | 699,247 ( 85 ) | 1,129 | 91.6 |
| Background | 2 | ATGGCTT | 0 | 686,114 | 589,877 ( 86 ) | 1,230 | 97.7 |
| Background | 2 | ATGGCTT | 1 | 687,440 | 592,155 ( 86 ) | 1,247 | 98.0 |
| Background | 2 | ATGGCTT | 2 | 687,684 | 590,399 ( 86 ) | 1,250 | 99.9 |
| Background | 2 | ATGGCTT | 3 | 687,023 | 588,474 ( 86 ) | 1,240 | 99.1 |
| Background | 6 | GCATAGT | 0 | 780,732 | 688,823 ( 86 ) | 1,215 | 97.2 |
| Background | 6 | GCATAGT | 1 | 780,587 | 688,530 ( 86 ) | 1,218 | 96.0 |
| Background | 6 | GCATAGT | 2 | 780,158 | 689,854 ( 86 ) | 1,262 | 100.3 |
| Background | 6 | GCATAGT | 3 | 780,935 | 688,767 ( 86 ) | 1,247 | 97.9 |
| Background | 9 | TGCAACT | 0 | 799,625 | 695,704 ( 83 ) | 1,260 | 99.4 |
| Background | 9 | TGCAACT | 1 | 799,355 | 687,400 ( 82 ) | 1,278 | 98.7 |
| Background | 9 | TGCAACT | 2 | 799,251 | 692,720 ( 83 ) | 1,279 | 100.5 |
| Background | 9 | TGCAACT | 3 | 798,652 | 686,969 ( 82 ) | 1,297 | 102.2 |
| Iron Reduction | 3 | CGAGATT | 0 | 801,054 | 691,322 ( 83 ) | 1,172 | 94.4 |
| Iron Reduction | 3 | CGAGATT | 1 | 800,915 | 692,221 ( 83 ) | 1,182 | 95.6 |
| Iron Reduction | 3 | CGAGATT | 2 | 801,326 | 694,750 ( 84 ) | 1,173 | 94.0 |
| Iron Reduction | 3 | CGAGATT | 3 | 801,701 | 693,653 ( 84 ) | 1,160 | 95.9 |
| Iron Reduction | 7 | ACTAGCT | 0 | 785,002 | 665,315 ( 85 ) | 1,229 | 98.6 |
| Iron Reduction | 7 | ACTAGCT | 1 | 786,124 | 665,021 ( 85 ) | 1,229 | 97.6 |
| Iron Reduction | 7 | ACTAGCT | 2 | 785,541 | 664,807 ( 85 ) | 1,228 | 99.2 |
| Iron Reduction | 7 | ACTAGCT | 3 | 785,449 | 665,049 ( 85 ) | 1,224 | 99.8 |
| Iron Reduction | 10 | TTGCGAT | 0 | 792,689 | 666,206 ( 85 ) | 1,227 | 98.4 |
| Iron Reduction | 10 | TTGCGAT | 1 | 792,157 | 670,043 ( 85 ) | 1,228 | 99.1 |
| Iron Reduction | 10 | TTGCGAT | 2 | 792,440 | 668,293 ( 85 ) | 1,210 | 96.1 |
| Iron Reduction | 10 | TTGCGAT | 3 | 792,952 | 668,844 ( 85 ) | 1,231 | 98.1 |
| Iron Reduction | 12 | GAGCAAT | 0 | 824,775 | 697,942 ( 83 ) | 1,166 | 95.7 |
| Iron Reduction | 12 | GAGCAAT | 1 | 825,566 | 698,680 ( 83 ) | 1,151 | 96.1 |
| Iron Reduction | 12 | GAGCAAT | 2 | 824,726 | 699,562 ( 83 ) | 1,139 | 95.1 |
| Iron Reduction | 12 | GAGCAAT | 3 | 825,102 | 697,696 ( 83 ) | 1,177 | 98.1 |
| Sulfate Reduction | 4 | ACACTGT | 0 | 833,592 | 672,744 ( 84 ) | 1,181 | 100.1 |
| Sulfate Reduction | 4 | ACACTGT | 1 | 833,809 | 673,495 ( 84 ) | 1,161 | 99.2 |
| Sulfate Reduction | 4 | ACACTGT | 2 | 832,899 | 671,761 ( 84 ) | 1,134 | 96.5 |
| Sulfate Reduction | 4 | ACACTGT | 3 | 833,229 | 672,694 ( 84 ) | 1,148 | 97.4 |
| Sulfate Reduction | 5 | CATTCGT | 0 | 829,719 | 671,369 ( 85 ) | 1,097 | 96.6 |
| Sulfate Reduction | 5 | CATTCGT | 1 | 830,245 | 667,095 ( 84 ) | 1,161 | 95.5 |
| Sulfate Reduction | 5 | CATTCGT | 2 | 830,014 | 668,843 ( 84 ) | 1,110 | 95.3 |
| Sulfate Reduction | 5 | CATTCGT | 3 | 830,436 | 672,422 ( 85 ) | 1,136 | 97.0 |
| Sulfate Reduction | 8 | CAGTACT | 0 | 843,002 | 684,684 ( 82 ) | 1,178 | 98.4 |
| Sulfate Reduction | 8 | CAGTACT | 1 | 843,139 | 680,442 ( 81 ) | 1,174 | 99.2 |
| Sulfate Reduction | 8 | CAGTACT | 2 | 842,217 | 684,541 ( 82 ) | 1,195 | 99.3 |
| Sulfate Reduction | 8 | CAGTACT | 3 | 842,777 | 688,275 ( 82 ) | 1,188 | 98.7 |
| Sulfate Reduction | 11 | GCTACAT | 0 | 836,132 | 697,663 ( 85 ) | 1,132 | 97.8 |
| Sulfate Reduction | 11 | GCTACAT | 1 | 835,464 | 696,991 ( 84 ) | 1,160 | 97.7 |
| Sulfate Reduction | 11 | GCTACAT | 2 | 836,482 | 698,612 ( 85 ) | 1,140 | 96.1 |
| Sulfate Reduction | 11 | GCTACAT | 3 | 835,853 | 699,251 ( 85 ) | 1,174 | 98.6 |
