# Supplementary material for: Short-Read Assembly of Full-Length 16S Amplicons Reveals Bacterial Diversity in Subsurface Sediments
Source: PLoS One. 2013 Feb 6;8(2):e56018. doi: 10.1371/journal.pone.0056018 (PMC3566076; doi:10.1371/journal.pone.0056018)
Supplement: Methods S1 — Additional details of sample collection, DNA extraction, amplification, sequencing, and analysis. (DOC) [file pone.0056018.s007.doc]

Short-read assembly of full-length 16S amplicons reveals bacterial diversity in subsurface sediments

Christopher S. Miller, Kim M. Handley, Kelly C. Wrighton, Kyle R. Frischkorn, Brian C. Thomas, Jillian F. Banfield

# Supporting Methods S1

## Sample collection, DNA extraction

Sediment was retrieved from a previously un-amended portion of the Rifle aquifer (Department of Energy, Integrated Field Research Challenge site, Colorado), sieved to remove large particles > 2mm, and packed into flow-through columns as described previously (Handley *et al.*, 2012). Columns (4 cm inner diameter, and 12 cm long) were placed into a freshly-drilled groundwater well and equilibrated with subsurface conditions for >1 week. After this period, groundwater was pumped up through the columns at 36 µl min^-1^, and influent groundwater tubes were amended with excess acetate (average final concentration in column effluent >4 mM) by pumping an anoxic groundwater solution containing up to 15 mM sodium acetate up through the columns (bottom → top). Columns were collected at three time points: (1) prior to acetate-amendment; and (2) after 1 week and (3) after 6.5 weeks of acetate-amendment, when paired geochemical data showed an increase in aqueous Fe(II) and decrease in sulfate concentrations, respectively (Handley KM, Wrighton KC, Miller CS, Wilkins MJ, Kantor RG, Williams KH, Long PE, Banfield JF, unpublished data). Sediment was homogenized and flash frozen upon collection.

Genomic DNA was extracted from a 5 g of sediment from each sample using PowerMax® Max Soil DNA Isolation Kits (MoBio Laboratories, Inc., Carlsbad, CA, USA) with the following modification to the manufacturer’s instructions. Sediment was vortexed at maximum speed for an additional 2 mins in the SDS reagent, and then incubated for 30 mins at 60°C in place of extended bead beating. The eluted volume was 5 ml per tube, as per the manufacturer’s instructions. DNA was concentrated by ethanol precipitation with glycogen.

## PCR amplification and sequencing

DNA was amplified using the universal bacterial 16S rRNA gene primers 27F (5’-AGAGTTTGATCCTGGCTCAG-3’) and 1492R (5’-GGTTACCTTGTTACGACTT-3’) (Lane, 1991), and temperature gradient PCR in order to minimize PCR bias. Reactions contained 5 units µl^-1^ of *TaKaRa Ex Taq*^TM^ (Takara Bio Inc., Otsu, Japan), and conditions were as follows: DNA was denatured for 1 min at 94°C; followed by 30 cycles consisting of denaturation for 1 min at 94°C, annealing for 30 s at 48-58°C (8 temperature gradient), and extension for 1 min at 72°C; and a final extension for 7 min at 72°C. Amplicons from the same sample were pooled, precipitated and quantified as described above. Fragment size and quality was checked by gel electrophoresis. For the spike-in control sample, we used 25 PCR cycles and 11 different annealing temperatures.

PCR products across the temperature gradient were pooled for each biological sample. Illumina library preparation and sequencing followed standard protocols at the University of California Davis DNA Technologies Core Facility (http://dnatech.genomecenter.ucdavis.edu). For each biological sample four replicate libraries were made. Briefly, amplicon DNA was fragmented to an expected average size of 300bp (actual range of mean insert size: 251 bp - 299 bp) using the Bioruptor NGS (Diagenode), and sheared fragments were used as input into an automated library preparation protocol on the on the Appollo 324 robot (Integenx) following the manufacturer's instructions. Each of 12 unique barcodes consisted of 7 nucleotides incorporated downstream of the sequencing primer. Barcodes were incorporated in both adapters ligated to each fragment. Adapter-ligated fragments were enriched with 12 cylces of PCR before library quantification and validation. Libraries were pooled in equal amounts and sequenced on the Illumina HiSeq 2000 using paired-end sequencing with 100 cycles (93 bp reads after removing indices), and processed with Casava verison 1.7. The spike-in control sample was sequenced with 101 bp reads, using an barcode internal to the adapter read as a separate indexing read, and was processed (including demultiplexing) with Casava version 1.8.2. Raw read data is deposited in the NCBI Short Read Archive (accession numbers SRX156410 - SRX156422). A table of read membership of the subsamples described in the manuscript is available for download at http://genegrabber.berkeley.edu/EMIRGE

## Read demultiplexing, trimming, and subsample dataset creation

Identical indexing barcodes identifying each library were incorporated as the first 7 basepairs of read 1 and read 2 (Table 1). To bin reads into the appropriate library, we calculated the hamming distance between each barcode and the set of 12 barcodes. If this distance was less than 3, we binned the read pair to the barcode with the lowest distance, discarding read pairs that were equidistant to two or more barcodes. Barcodes were removed, leaving 93 bp reads. The spike in control utilized newer Illumina adapters with an internal index (see above), thus the full 101 bp reads were used. For each library, reads were sampled at random without replacement into four separate 1 million read subsamples. Reads from each subsample were trimmed back from the 3’ end to remove bases with a quality score of 2 or lower, and read pairs were discarded if one or both reads was shorter than 75 bp.

## Calculations of expected coverage

To calculate expected coverage in the middle of a sequence, we assumed an average sequence length (from EMIRGE-reconstructed sequences) of 1464 bp. For a data set of one million reads, we expect 100 reads to come from a rRNA sequence of relative abundance 0.001. From our empirically determined probabilities for start sites (see main text and Figure 1), we calculated that, on average, a read starts within 100 bp of an amplicon end 25.05% of the time, leaving 75 of the 100 reads to start in the “middle” of the sequence. Assuming these *N* reads of length *L* start at random in the middle of a gene (of length *G* = 1264 bp) with roughly uniform probability (Figure 1), coverage (C) can be calculated as:

$C=\frac{L*N}{G}=\frac{(93*2)*75}{1264}=11.04$.

In a Poisson model, coverage is equal to the parameter λ and allows for calculation of the probability that a base is sequenced with some minimum coverage (Lander & Waterman, 1988) . Results were similar under a binomial model of fragment starts. Other subsample datasets examined followed similar patterns.

# Supplementary Data

Additional supplementary data files are available at:

http://banfieldlab.berkeley.edu/EMIRGE/

EMIRGE_OTU_abundances.txt

This file contains all EMIRGE OTU estimated abundances for each of the 48

subsample datasets in tab-delimited text. Also included are RDP classifier

and SILVA Blast-based assignments of taxonomy.

EMIRGE_reconstructed_16S.fasta

This file contains all EMIRGE-reconstructed 16S rRNA sequences with

abundance >= 0.01 % for each of the 48 subsample datasets. Each record

specifies the index (iXX) and subsample (sX) in the header.

EMIRGE_reconstructed_16S_clustered_OTUs.txt

This file contains a tab-delimited mapping from clustered OTU to the

EMIRGE-reconstructed sequences contained within that cluster.

EMIRGE_reconstructed_16S_spike_in_control.fasta

This file contains all EMIRGE-reconstructed 16S rRNA sequences with

abundance >= 0.01 % for the spike in-control dataset.

trimmed_subsampled_reads.txt

This tab-delimited file contains 3 columns mapping the reads (available at

the NCBI Sequence Read Archive under accession SRA054986) to the

subsamples described in the manuscript. Columns are : 1. index (barcode)

2. subsample and 3. read name.
